# Automatic processing of facial affects in patients with borderline personality disorder: associations with symptomatology and comorbid disorders

**DOI:** 10.1186/s12991-015-0058-y

**Published:** 2015-07-14

**Authors:** Uta-Susan Donges, Bibiana Dukalski, Anette Kersting, Thomas Suslow

**Affiliations:** Department of Psychosomatic Medicine, University of Leipzig, Leipzig, Germany; Department of Psychiatry, University of Münster, Münster, Germany

**Keywords:** Borderline personality disorder, Affective priming, Automatic processing, Perception, Facial expression, Anger, Evaluative judgment, Attention allocation

## Abstract

**Background:**

Instability of affects and interpersonal relations are important features of borderline personality disorder (BPD). Interpersonal problems of individuals suffering from BPD might develop based on abnormalities in the processing of facial affects and high sensitivity to negative affective expressions. The aims of the present study were to examine automatic evaluative shifts and latencies as a function of masked facial affects in patients with BPD compared to healthy individuals. As BPD comorbidity rates for mental and personality disorders are high, we investigated also the relationships of affective processing characteristics with specific borderline symptoms and comorbidity.

**Methods:**

Twenty-nine women with BPD and 38 healthy women participated in the study. The majority of patients suffered from additional Axis I disorders and/or additional personality disorders. In the priming experiment, angry, happy, neutral, or no facial expression was briefly presented (for 33 ms) and masked by neutral faces that had to be evaluated. Evaluative decisions and response latencies were registered. Borderline-typical symptomatology was assessed with the Borderline Symptom List.

**Results:**

In the total sample, valence-congruent evaluative shifts and delays of evaluative decision due to facial affect were observed. No between-group differences were obtained for evaluative decisions and latencies. The presence of comorbid anxiety disorders was found to be positively correlated with evaluative shifting owing to masked happy primes, regardless of baseline—neutral or no facial expression condition. The presence of comorbid depressive disorder, paranoid personality disorder, and symptoms of social isolation and self-aggression were significantly correlated with response delay due to masked angry faces, regardless of baseline.

**Conclusions:**

In the present affective priming study, no abnormalities in the automatic recognition and processing of facial affects were observed in BPD patients compared to healthy individuals. The presence of comorbid anxiety disorders could make patients more susceptible to the influence of a happy expression on judgment processes at an automatic processing level. Comorbid depressive disorder, paranoid personality disorder, and symptoms of social isolation and self-aggression may enhance automatic attention allocation to threatening facial expressions in BPD. Increased automatic vigilance for social threat stimuli might contribute to affective instability and interpersonal problems in specific patients with BPD.

## Background

The main characteristic of borderline personality disorder (BPD) is a pervasive pattern of instability in interpersonal relations, identity, and affects as well as impulsivity that begin by early adulthood [1, 2]. According to the biosocial model of Linehan [3], the interpersonal problems of individuals suffering from BPD develop due to a high sensitivity and reactivity to affective stimuli and difficulties in down-regulation of affective reactions. It has been argued that adverse childhood experiences might play an important role in the development of intense fears of abandonment and deficits in trust and cooperation, which are frequently observed in people with BPD [4, 5]. There is evidence that individuals with BPD experience more negative affect and anger in response to interpersonal stressors than healthy participants [6].

A prerequisite for successful social interaction and harmonious relationships is the ability to correctly recognize nonverbal interpersonal cues and especially affective facial expressions of others. Facial expressions of affects convey important information about feeling states, intentions, wishes, and beliefs to persons in the environment [7, 8]. Against this background, it is not surprising that much research has been conducted in the last few years examining the perception of facial affect in individuals with BPD. This field of research should increase our understanding of basic perceptual factors possibly underlying dysfunctional interaction styles [9] and define more precisely the sensitivities and biases in the perception of others’ facial affects with respect to BPD [10].

To date, no study has examined automatic affective judgments based on facial expression in those with BPD. According to Zajonc [11], affective reactions can be elicited by minimal stimulus input and have judgmental and physiological consequences. Researchers have repeatedly employed masked presentations of affective facial expressions to assess basic affective reactions [12, 13]. It has been determined that valence of briefly presented facial expression systematically influences judgments of subsequent (neutral or ambiguous) stimuli [12]. For instance, stimuli preceded by happy faces were evaluated more positively than those preceded by neutral expressions, whereas stimuli preceded by angry faces were evaluated more negatively than those preceded by neutral primes. The phenomenon of affect-congruent influence of facial expression on subsequent judgments is referred to as the “affective priming effect” [12]. Non-conscious processing of facial affects can influence decision making [14] and can have rather long-lasting effects on memory [15].

The aims of the present study were to examine affective priming (i.e., automatic evaluative shifts) owing to masked facial affects in patients with BPD compared to healthy individuals and to investigate the relationship of affective priming with specific borderline symptoms and comorbidity. Borderline-typical symptomatology was assessed according to the Borderline Symptom List (BSL [16]). BPD comorbidity rates for Axis I and II disorders are high. In fact, even in community samples, BPD is rarely diagnosed alone [17]. Individuals with a BPD diagnosis are especially likely to have co-occurring depressive and anxiety disorders [17] as well as paranoid, avoidant, dependent, and/or obsessive–compulsive personality disorders [18]. Our patient recruitment strategy was inclusive, and we controlled for comorbidity effects through post hoc analyses. Although co-occurrence of disorders certainly increases diagnostic noise, it also renders results more generalizable. However, BPD patients with current substance abuse or dependence or with bipolar or psychotic disorders were excluded.

We analyzed the affective priming data taking into consideration evaluative judgments and response latencies. Changes in evaluative scores due to affective primes reflect shifts in evaluative decisions compared to neutral primes, whereas changes in response latencies owing to affective primes reflect the slowing or speeding up of evaluative decisions compared to neutral primes. In the present experiment, angry, happy, neutral, or no facial expressions were briefly presented and masked by neutral faces. Participants had to evaluate the neutral mask face. Only women participated in the current study. This decision was based on the facts that BPD is diagnosed predominantly (approximately 75%) in women [1] and women have shown stronger affective priming effects than men [19].

It was hypothesized that BPD patients manifest stronger evaluative shifts owing to angry facial expression compared to healthy individuals. Moreover, we expected that BPD patients would exhibit more response delay due to angry primes compared to healthy controls. That is, we assumed that BPD patients’ evaluative judgments are more negatively affected by masked facial anger expressions and their evaluative decisions are more disrupted by angry expressions than those of healthy persons. As impaired recognition of affective facial and prosodic stimuli has been found to be associated with interpersonal antagonism in BPD [20], it was assumed that hostility of BPD patients is related to less affective priming (i.e., evaluative shifts). Results from a recent affective priming study suggest that social anxiety is correlated with stronger positive priming effects due to masked happy faces [21]. Against this background, it was hypothesized that the presence of comorbid anxiety disorders is associated with enhanced evaluative shifts due to happy facial primes in BPD patients.

To assess the success of masking prime stimuli, study participants were interviewed after the priming task and asked what they noticed during the experiment. As BPD patients tend to manifest negative response biases [22], we expected that, when asked, BPD patients would report having seen more negative affect qualities (e.g., sadness, fear, or disgust) than healthy controls.

## Methods

### Participants

The study included 29 patients at the Department of Psychosomatic Medicine of the University of Leipzig who met the DSM-IV criteria for BPD and 38 healthy controls. All participants were women. The screening of healthy volunteers and psychiatric (DSM-IV Axes I and II) diagnoses of patients were assessed using the German language versions of the Structured Clinical Interview for DSM-IV (SCID-I) [23] and the SCID-II [24]. The majority of the BPD patients suffered from additional Axis I disorders (SCID-I) and/or additional personality disorders (SCID-II) (see below). For all participants, exclusion criteria included a history of neurological disease, and current substance abuse or dependence. Subjects were required to be between 18 and 39 years of age, to have normal or corrected-to-normal vision and to have German as their first language. Patients did not have (actual or lifetime) bipolar or psychotic disorders. Healthy subjects were free of any lifetime history of psychiatric disorders. Moreover, no healthy participant had a BDI-II score indicative of moderate or severe depression (>12). Table 1 lists the demographic and questionnaire data for all participants.

**Table 1:** Demographic characteristics, intelligence, and affectivity of study groups, borderline symptomatology and comorbid diagnoses of patients

Variable	BPD patients (*n* = 29)	Healthy subjects (*n* = 38)
	Mean (SD)	Mean (SD)
Age (years)	27.7 (5.9)	24.3 (4.0)
Education (years)	11.2 (1.5)	12.4 (0.6)
% Married/partnership	24 (7)	58 (22)
Intelligence (IQ, MWT-B)	106.1 (11.2)	115.0 (12.4)
Depression (BDI-II)	21.3 (10.8)	5.9 (3.0)
Trait anxiety (STAI)	63.1 (9.0)	36.0 (6.0)
BST		
Self-perception	0.98 (0.83)	
Affect regulation	1.71 (0.82)	
Self-destruction	1.19 (1.08)	
Dysphoria	2.59 (0.67)	
Loneliness	1.30 (0.81)	
Hostility	1.34 (0.64)	
Intrusions	0.65 (0.60)	
Total	1.41 (0.69)	
Axis I disorders		
% Affective	24 (7)^a^	
% Anxiety	38 (11)	
% Somatoform	31 (9)	
% Eating	28 (8)	
Personality disorders		
% Paranoid	34 (10)	
% Schizotypal	7 (2)	
% Avoidant	65 (19)	
% Dependent	17 (5)	
% Obsessive–compulsive	38 (11)	

*MWT-B* multiple choice vocabulary test, *STAI-Trait* State–Trait Anxiety Inventory, trait version, *BDI-II* Beck Depression Inventory, *BSL* Borderline Symptom List.

^a^The number in parentheses specifies the absolute number of patients.

As previously mentioned, several BPD patients were noted to have comorbid mental disorders. Seven patients suffered from affective disorders (major depression: *n* = 6, dysthymia: *n* = 1). Eleven patients had evidence of anxiety disorders (panic disorder: *n* = 6, social phobia: *n* = 3, simple phobia: *n* = 1, posttraumatic stress disorder: *n* = 2). Nine patients suffered from somatoform disorders (body dysmorphic disorder: *n* = 5, pain disorder: *n* = 1, somatization disorder: *n* = 3). Eight patients suffered from bulimia nervosa. With regard to Axis II disorders, 19 patients fulfilled the diagnostic criteria for avoidant personality disorder. Eleven patients had evidence of an obsessive–compulsive personality disorder. Ten patients fulfilled the diagnostic criteria for paranoid personality disorder. Five patients had evidence of dependent personality disorder and two patients met the criteria for schizotypal personality disorder. The vast majority of patients were taking antidepressants—selective serotonin reuptake inhibitors.

With regard to age and education, patients differed from control subjects. According to the results of unpaired *t* tests, patients were older [*t*(65) = 2.81, *p* < 0.01] and less educated than control subjects [*t*(65) = −4.60, *p* < 0.001]. As could be expected, healthy participants were more frequently married or had stable non-marital partners [*χ*^2^(1) = 7.63, *p* < 0.01] (see Table 1).

The present study was carried out according to the Declaration of Helsinki [25], and written informed consent was obtained from all subjects. The study was approved by the ethics committee of the Medical Faculty of the University of Leipzig. All participants received financial compensation after completing the study.

### Psychometric instruments

In our study, intelligence was assessed using the multiple choice vocabulary test (MWT-B [26]). The German versions of the Beck-Depression Inventory (BDI-II [27]) and the State–Trait Anxiety Inventory (STAI [28]) were administered to measure participants’ depressive state and trait anxiety (see Table 1 for details). In the patient sample, the Borderline Symptom List (BSL), which consists of seven subscales, self-perception, affect regulation, self-destruction, dysphoria, loneliness, hostility, and intrusions, was administered to assess borderline-typical symptomatology [16, 29]. The BSL has demonstrated good psychometric properties in several studies [16, 29]. The BSL subscale and total scores in our sample (see Table 1) were somewhat lower than those found in other studies for BPD patients.

### Affective priming task: stimulus material and procedure

**Figure 1:**
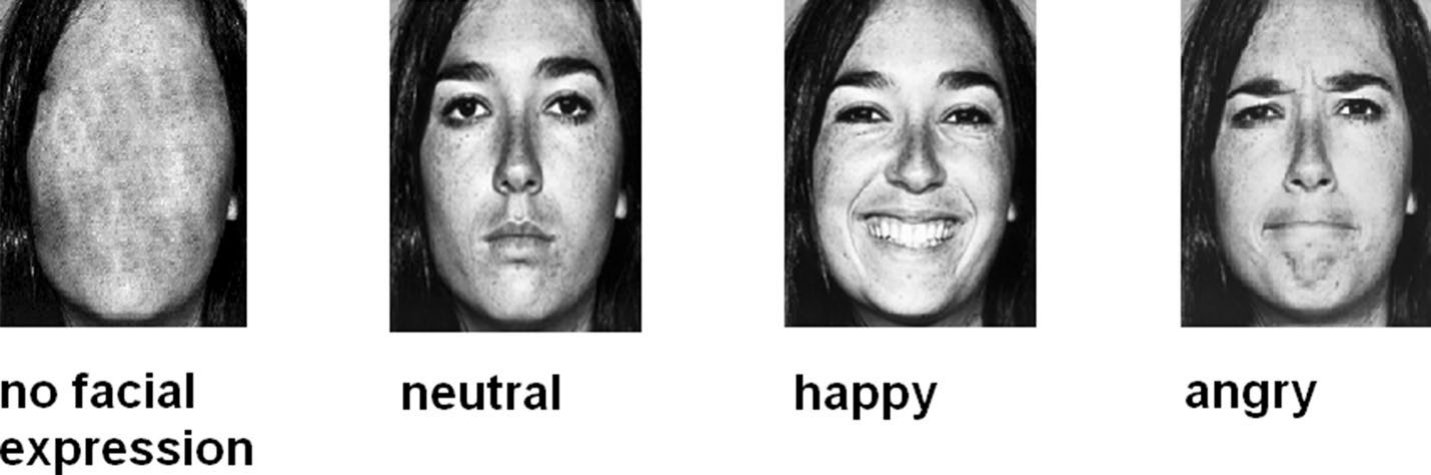
Examples of faces presented in the four prime conditions—happy, angry, neutral, and no facial expression. Faces were accessed from the Pictures of Facial Affect database provided by Ekman and Friesen [30].

In our affective priming experiment, stimuli consisted of monochrome angry, happy, and neutral expressions accessed from the Pictures of Facial Affect database [30]. Affective and neutral faces of ten individuals (50% female for each facial type) were applied as primes. To avoid identity of prime and mask stimulus in the neutral prime condition, neutral primes were vertically mirrored. That is, neutral prime faces were created by a mirror inversion (left to right) of neutral mask faces. In our affective priming task, 80 trials were presented: 20 with angry, 20 with happy, and 20 with neutral prime faces. In 20 trials, primes with no facial features were shown. Thus, each trial was presented twice. In each trial, facial expressions of the same individual were displayed. In the no facial expression condition, stimuli consisted of neutral faces in which central facial features—mouth, nose, and eyes—had been replaced by a surface without contours (see Figure 1 for examples of prime stimuli). Trials were presented in a fixed random sequence with the constraints that no two subsequent trials depicted the same person, not more than two subsequent trials showed the same prime category, and no trial was shown twice per half.

**Figure 2:**
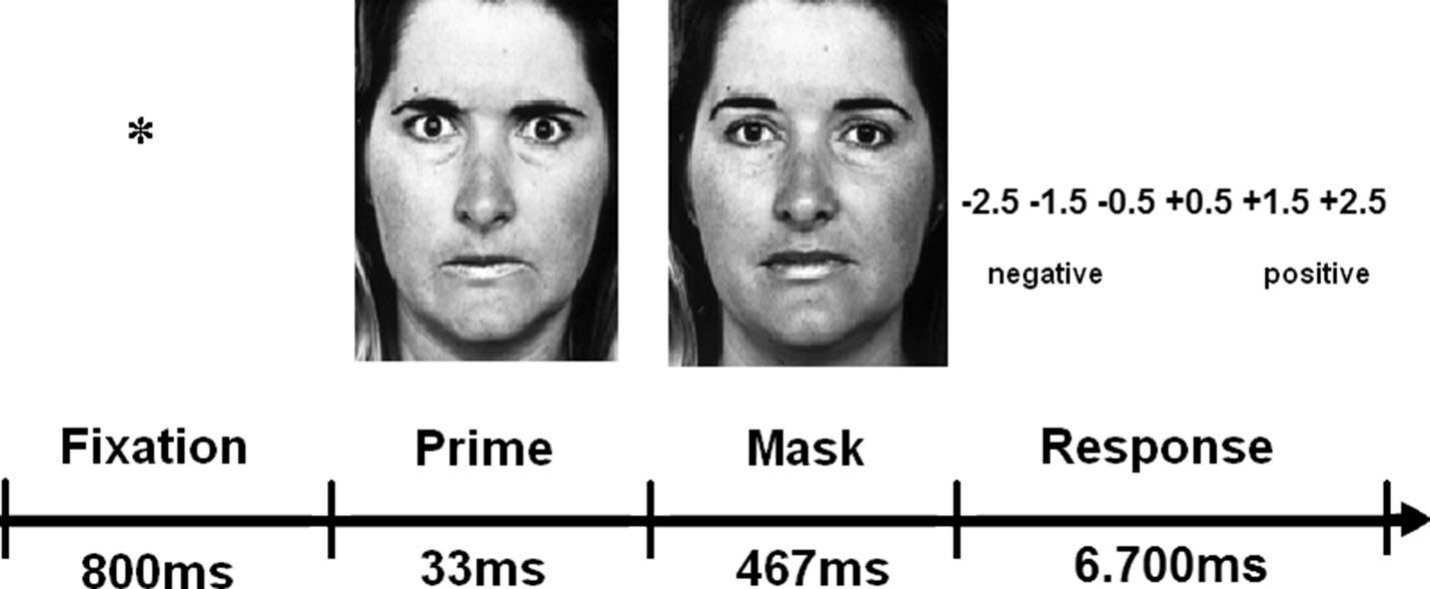
Sequence of events within trials in the affective priming experiment. Participants were instructed to view a series of faces and evaluate the expressions as negative or positive on a six-point scale ranging from −2.5 to +2.5 by pressing a button on the keyboard. In our example, a trial with an angry prime face is shown. Faces were accessed from the Pictures of Facial Affect database provided by Ekman and Friesen [30].

Participants were instructed to view a series of faces and evaluate the expression as negative or positive on a six-point scale ranging from −2.5 to +2.5 by pressing a button on the keyboard. Each trial had a duration of 8 s, in which a prime face was shown for 33 ms preceded by a fixation cross displayed for 800 ms and followed by a neutral face that was shown for 467 ms. This was then followed by a blank screen for 6.700 ms (Figure 2). The affective priming experiment had an overall duration of 10 min and 40 s. The computer-based stimulus presentation and response registration were realized via the Inquisit program [31] on a Dell Latitude E6500 with a monitor refresh rate of 60 Hz.

### Statistical analysis

Mean evaluative ratings and mean response latencies were determined for each study group and prime condition (see Tables 2, 3). First, the affective priming data were analyzed in a repeated measures analysis of variance (ANOVA), with one between-subjects factor (group: patient vs. healthy subjects) and one within-subjects factor (prime face: happy, angry, neutral, and no facial expression). ANOVAs were conducted separately for evaluative and reaction time data.

**Table 2:** Evaluative responses to neutral mask faces as a function of prime and study group

	BPD (*n* = 29)	Healthy (*n* = 38)
	Mean (SD)	Mean (SD)
Angry prime	−0.266 (0.490)	−0.193 (0.337)
Happy prime	−0.149 (0.512)	−0.086 (0.405)
Neutral prime	−0.224 (0.425)	−0.107 (0.325)
No facial expression	−0.192 (0.454)	−0.128 (0.328)

**Table 3:** Response latencies (in ms) as a function of prime and study group

	BPD (*n* = 29)	Healthy (*n* = 38)
	Mean (SD)	Mean (SD)
Angry prime	1,693 (449)	1,706 (380)
Happy prime	1,657 (480)	1,700 (383)
Neutral prime	1,587 (438)	1,619 (377)
No facial expression	1,577 (426)	1,642 (364)

In addition, two affective priming scores were calculated for angry and happy faces by using neutral faces and no facial expressions as baseline conditions. These priming scores were used in the subsequent correlation analyses. Affective priming for angry and happy faces was computed by subtracting mean evaluative ratings for neutral mask faces primed by neutral faces (or no facial expression) from mean evaluative ratings for neutral mask faces primed by happy (or angry) faces. A positive priming score for happy faces indicates that participants rated neutral masks more positively if they were primed by happy faces compared with neutral masks primed by neutral faces (see Table 4 for priming scores). A negative priming score for angry faces indicates that participants evaluated the neutral masks more negatively if they were primed by angry faces compared with neutral masks primed by neutral faces. The same calculations were performed for the reaction time data (see Table 5 for latency difference scores). One sample *t* test was used to determine whether the priming scores and the latency difference scores were different from zero.

**Table 4:** Affective priming scores based on angry and happy primes as a function of the study group (baseline conditions: neutral primes and no facial expression)

	BPD (*n* = 29)	Healthy (*n* = 38)
	Mean (SD)	Mean (SD)
Angry prime (neutral face baseline)	−0.042 (0.215)	−0.085 (0.164)
Angry prime (no face baseline)	−0.074 (0.230)	−0.064 (0.175)
Happy prime (neutral face baseline)	0.075 (0.233)	0.022 (0.255)
Happy prime (no face baseline)	0.043 (0.230)	0.042 (0.260)

**Table 5:** Latency difference scores for angry and happy prime conditions (in ms) as a function of the study group (baseline conditions: neutral primes and no facial expression)

	BPD (*n* = 29)	Healthy (*n* = 38)
	Mean (SD)	Mean (SD)
Angry prime (neutral face baseline)	106 (168)	86 (161)
Angry prime (no face baseline)	115 (159)	65 (133)
Happy prime (neutral face baseline)	70 (172)	81 (196)
Happy prime (no face baseline)	80 (155)	59 (190)

Product–moment and Spearman rank correlation analyses were performed to investigate the relationships of priming scores (and latency difference scores) with demographic variables, intelligence, affectivity, borderline symptoms, and comorbidity (presence and number of Axis I and Axis II comorbidities) in the patient sample and/or in the whole study sample. The Chi square test was used to test for an association between the study group and subjective prime awareness. If not otherwise specified, the results were considered significant at *p* = 0.05, two tailed.

### Awareness check

To evaluate the success of masking facial expressions, participants were asked immediately after the priming task whether they had noticed anything out of the ordinary and whether they had perceived anything just prior to the neutral target faces. The subjective threshold is the oldest criterion for demonstrating perception without awareness and is preferred because it directly assesses the conscious experience of subjects [32]. If participants stated that they had noticed faces or affective expressions that were shown before the neutral (target) faces, they were questioned whether they had perceived faces expressing sadness, surprise, fear, happiness, anger, or disgust.

### General procedure

After consenting to participate, a clinical diagnostic interview was conducted. In this first session, the SCID I and SCID II were administered, demographic data were registered, and patients completed the BSL. All participants were tested individually. A few days later, during a second session, participants were administered the MWT-B, the BDI-II, and the STAI in a fixed order. After a short break, the affective priming experiment was conducted. Testing sessions were administered in a quiet room that was free from auditory and visual distractions. The computer monitor was placed directly in front of the participant with the participant’s eyes approximately 60 cm from the screen. Participants were instructed to keep their fingers close to the response buttons during the experiment. Immediately after the priming task, the subjective awareness check was conducted. Having completed the tests, participants were fully debriefed about the experimental procedure, given the opportunity to ask questions and thanked for their assistance.

## Results

### Psychometric instruments: between-group comparison

As could be expected, BPD patients had higher depression (BDI-II) and trait anxiety (STAI) scores than healthy individuals [*t*(65) = 8.39, *p* < 0.001; *t*(65) = 14.81, *p* < 0.001]. Moreover, BPD patients recorded lower intelligence scores (MWT-B) compared to healthy controls [*t*(65) = −3.10, *p* < 0.01]. However, depression and trait anxiety were not associated with priming or latency difference parameters in the whole sample (see below). Intelligence correlated, instead, with latency differences based on angry faces (compared to neutral prime baseline), but it did not correlate with evaluative priming scores (see below for details).

### Awareness check

Thirteen of 29 patients and 25 of 38 healthy individuals reported to have noticed faces or affective expressions that were shown prior to the neutral faces. Global subjective awareness of primes was not associated with the study group [*χ*^2^(1) = 2.94, *p* > 0.05]. None of our study participants affirmed to have seen happy and angry faces before the neutral faces and further denied having seen faces expressing sadness, surprise, fear, or disgust. Thus, none of the participants showed awareness responses suggesting perfect identification and discrimination of affective primes. Interestingly, BPD patients and healthy individuals did not differ in reporting perceptions of happy, surprised, and disgusted faces (*p* > 0.05), but BPD patients more frequently perceived anger [*χ*^2^(1) = 4.03, *p* < 0.05], fear [*χ*^2^(1) = 10.20, *p* < 0.001], and sadness [*χ*^2^(1) = 4.53, *p* < 0.05]. The latter response behavior suggests a bias toward the perception of threat-related or negative facial expressions in BPD patients.

### Affective priming task

There was a general tendency in our sample to evaluate neutral mask faces on average as rather negative (see Table 2). According to the results of a repeated measures ANOVA of evaluative responses with one between-subjects factor (group: BPD patients vs. healthy individuals) and one within-subjects factor (prime face: happy, angry, neutral, and no facial expression), there was a significant effect of prime face [*F*(3, 63) = 4.13, *p* < 0.01, partial *η*^2^ = 0.17], but no effect of group [*F*(1, 65) = 0.71, *p* = 0.40] or interaction between group and prime categories [*F*(3, 63) = 0.65, *p* = 0.58]. The results from paired *t* tests showed that evaluations in the angry prime condition differed significantly from those in the happy [*t*(66) = −3.35, *p* ≤ 0.001], neutral [*t*(66) = −2.90, *p* < 0.01], and no [*t*(66) = −2.82, *p* < 0.01] facial expression prime condition. No other significant results were observed.

A repeated measures ANOVA based on response latencies, with group as a between-subjects factor and prime condition as within-subjects factor, yielded a significant effect of prime face [*F*(3, 63) = 13.45, *p* < 0.001, partial *η*^2^ = 0.39] but no effect of group [*F*(1, 65) = 0.16, *p* = 0.69] or interaction between group and prime category [*F*(3, 63) = 0.66, *p* = 0.58] (see Table 3 for response latencies). According to the results from paired *t* tests, reaction times in the angry prime and the happy prime conditions differed significantly from those in the neutral and the no facial expression prime conditions (*p*s ≤ 0.005, respectively). Response latencies in the angry prime condition did not differ from those in the happy prime condition [*t*(66) = 0.80, *p* = 0.43]. Finally, response latencies in the no prime condition did not differ from those in the no facial expression condition [*t*(66) = −0.40, *p* = 0.69].

As noted, affective priming scores were calculated for evaluative and response latency data (see Tables 4, 5). Results from one sample *t* tests showed that the evaluative priming scores based on angry faces differed significantly from zero regardless of baseline condition [*t*(66) = −2.90, *p* < 0.01 (neutral prime baseline); *t*(66) = −2.82, *p* < 0.01 (no facial expression baseline)]. Thus, masked angry prime faces produced negative evaluative shifts. For happy primes, no significant priming effects were observed. However, using one-tailed testing shows some evidence of a prime valence-congruent shift in evaluative ratings owing to masked happy faces [*t*(66) = 1.49, *p* < 0.10, one-tailed (neutral prime baseline); *t*(66) = 1.42, *p* < 0.10, one-tailed (no facial expression baseline)]. In other words, neutral mask faces preceded by happy primes tended to be judged more positively than mask faces preceded by neutral or no facial expression primes.

Response latency difference scores based on angry faces differed significantly from zero regardless of baseline condition [*t*(66) = 4.75, *p* < 0.001 (neutral prime baseline); *t*(66) = 4.87, *p* < 0.001 (no facial expression baseline)] (see Table 5). Thus, it is concluded that the presentation of masked angry primes delayed evaluative responses. There were also significant effects for the happy prime condition [*t*(66) = 3.39, *p* < 0.01 (neutral prime baseline); *t*(66) = 3.19, *p* < 0.01 (no facial expression baseline)], thus indicating that the presentation of masked happy primes led also to increased response times compared to the presentation of neutral or no facial expression primes.

### Relationship of affective priming with demographic variables, intelligence, and affectivity: whole sample vs. patient sample

Product–moment correlation analyses were conducted to examine the relationships of priming scores with demographic variables, intelligence (MWT-B IQ), and affectivity (BDI-II, STAI). In the whole sample, there were no significant correlations between evaluative priming scores and demographic variables, intelligence, or affectivity. However, with respect to response latency difference scores, a correlation between intelligence and latency difference based on angry faces (baseline neutral primes) was found (*r* = −0.25, *p* < 0.05). This means that less delay in responding to masked angry primes was associated with higher intelligence. Moreover, depression was positively correlated with latency difference based on angry faces (no face baseline) (*r* = 0.28, *p* < 0.05) (see Table 6).

**Table 6:** Product–moment correlations between priming and latency difference scores and demographic variables, intelligence, and affectivity in the whole sample (*N* = 67)

Variables	Priming scores				Latency difference scores			
	A (ne)^a^	A (no)	H (ne)	H (no)	A (ne)	A (no)	H (ne)	H (no)
Age	0.00	−0.04	0.00	−0.03	0.02	−0.09	0.01	−0.09
Education	0.00	−0.07	0.12	0.06	−0.06	0.12	−0.15	0.00
Intelligence (IQ)	−0.11	0.00	−0.19	−0.12	−0.25*	−0.08	−0.17	−0.02
Depression (BDI)	0.02	0.05	−0.03	−0.01	0.14	0.28*	−0.01	0.09
Trait anxiety (STAI)	0.16	0.12	0.04	0.02	0.17	0.21	0.06	0.09

*A* (*ne*) angry prime vs. neutral prime baseline, *A* (*no*) angry prime vs. no facial expression baseline, *H* (*ne*) happy prime vs. neutral prime baseline, *H* (*no*) happy prime vs. no facial expression baseline.

^a^Experimental condition.

* *p* < 0.05.

In the patient sample, there were no significant correlations between evaluative priming scores and demographic variables, intelligence or affectivity. With respect to latency difference scores, two significant correlations were observed. Intelligence, as measured by the MWT-B, was negatively correlated with latency difference based on happy faces (*r* = −0.40, *p* < 0.05, baseline neutral prime condition). This means that a greater delay in responding in the masked happy prime condition was associated with less intelligence. Moreover, BDI-II scores were found to correlate positively with latency difference based on angry faces (*r* = 0.40, *p* < 0.05, baseline no facial expression condition) (see Table 7). Accordingly, depression was related to response inhibition owing to angry primes when compared with no facial prime.

**Table 7:** Product–moment correlations between priming and latency difference scores and demographic variables, intelligence, and affectivity in the patient sample (*N* = 29)

Variables	Priming scores				Latency difference scores			
	A (ne)^a^	A (no)	H (ne)	H (no)	A (ne)	A (no)	H (ne)	H (no)
Age	−0.01	−0.05	−0.20	−0.24	−0.01	−0.21	0.05	−0.14
Education	0.07	−0.06	0.19	0.06	−0.01	0.33	−0.16	0.17
Intelligence (IQ)	−0.14	−0.19	0.03	−0.03	−0.30	0.16	−0.40*	0.05
Depression (BDI)	−0.15	0.17	−0.24	0.07	0.27	0.40*	0.14	0.27
Trait anxiety (STAI)	0.10	0.36	−0.21	0.06	0.35	0.28	0.19	0.02

*A* (*ne*) angry prime vs. neutral prime baseline, *A* (*no*) angry prime vs. no facial expression baseline, *H* (*ne*) happy prime vs. neutral prime baseline, *H* (*no*) happy prime vs. no facial expression baseline.

^a^Experimental condition.

* *p* < 0.05.

### Relationship of affective priming with comorbidity and borderline symptomatology: results from the patient sample

Spearman rank correlations were calculated to investigate the relationship between priming and the presence of comorbid Axis I disorders (affective disorders, anxiety disorders, somatoform disorders, and bulimia nervosa) and Axis II disorders (avoidant personality disorder, obsessive–compulsive personality disorder, paranoid personality disorder, dependent personality disorder, and schizotypal personality disorder). In addition, the total number of comorbid Axis I and the total number of Axis II disorders were also considered in the correlation analysis (see Table 8 for details). The presence of anxiety disorders was related to stronger (evaluative) priming based on happy faces (*r* = 0.43, *p* < 0.05; neutral face baseline; *r* = 0.51, *p* < 0.01; no face baseline). Thus, suffering from an additional anxiety disorder was associated with more positive priming based on masked happy facial expressions. Obsessive–compulsive personality disorder was positively associated with the evaluative priming score based on angry faces (neutral face baseline) (*r* = 0.40, *p* < 0.05) and inversely associated with the evaluative priming score based on happy faces (no face baseline) (*r* = −0.39, *p* < 0.05). In addition, the presence of paranoid personality disorder was positively correlated with the evaluative priming score based on angry faces (no face baseline) (*r* = 0.42, *p* < 0.05). Thus, BPD patients with obsessive–compulsive and paranoid personality disorder evaluated neutral masks preceded by angry primes as more positive, which indicates prime valence-incongruent shifts, than patients without these personality disorders.

**Table 8:** Spearman rank correlations between priming and latency difference scores and comorbidity in the patient sample (*N* = 29)

Disorders	Priming scores				Latency difference scores			
	A (ne)^a^	A (no)	H (ne)	H (no)	A (ne)	A (no)	H (ne)	H (no)
Axis I disorders								
Affective	−0.15	0.04	−0.24	−0.08	0.32	0.45*	−0.01	0.12
Anxiety	−0.22	−0.06	0.43*	0.51**	0.04	−0.02	0.16	−0.03
Somatoform	−0.20	−0.34	−0.20	−0.11	−0.02	−0.31	0.16	−0.08
Eating	0.21	0.24	−0.11	−0.09	0.20	0.03	0.03	−0.23
Total number of Axis I disorders	−0.15	0.10	0.06	0.36	0.15	−0.24	0.32	−0.16
Personality disorders								
Paranoid	0.19	0.42*	−0.17	−0.01	0.43*	0.08	0.31	−0.09
Schizotypal	−0.15	0.04	−0.36	−0.28	−0.02	0.05	0.08	0.24
Avoidant	−0.06	0.09	−0.13	−0.17	−0.05	0.22	−0.27	−0.08
Dependent	−0.09	0.25	−0.18	0.14	0.17	0.05	0.15	0.01
Obsessive–compulsive	0.40*	0.02	−0.11	−0.39*	−0.05	−0.27	0.02	−0.46*
Total number of personality disorders	0.16	0.29	−0.32	−0.29	0.18	0.08	0.10	−0.13

*A* (*ne*) angry prime vs. neutral prime baseline, *A* (*no*) angry prime vs. no facial expression baseline, *H* (*ne*) happy prime vs. neutral prime baseline, *H* (*no*) happy prime vs. no facial expression baseline.

^a^Experimental condition.

** *p* < 0.01; * *p* < 0.05.

Correlation analysis concerning the latency difference scores revealed that the presence of depressive disorders was related to high latency difference scores in the angry prime condition (no prime baseline) (*r* = 0.45, *p* < 0.05). That is, the presence of affective disorders was associated with a delay in responding to angry primes. Moreover, paranoid personality disorder was also positively correlated with latency difference scores in the angry prime condition (neutral prime baseline) (*r* = 0.43, *p* < 0.05), and accordingly, the presence of paranoid personality disorder was associated with slower response times due to masked angry faces. Finally, the presence of obsessive–compulsive personality disorder was inversely associated with the latency difference scores in the happy prime condition (no face baseline) (*r* = −0.46, *p* < 0.05). There were no correlations of affective priming or latency difference scores with the total number of comorbid Axis I or the total number of Axis II disorders.

Product–moment correlation was applied to investigate the relationship of priming and latency difference scores with borderline symptomatology as assessed by the BSL (see Table 9 for details). The subscale Hostility positively correlated with the evaluative priming score based on angry faces (no face baseline). A negative correlation was observed between self-perception and intrusions and evaluative priming based on happy faces (neutral face baseline). This indicates that BPD patients with more intrusions and difficulties in self-perception manifested less positive priming owing to masked happy faces.

**Table 9:** Product–moment correlations between priming and latency difference scores and borderline symptomatology in the patient sample

BSL scales	Priming scores				Latency difference scores			
	A (ne)^a^	A (no)	H (ne)	H (no)	A (ne)	A (no)	H (ne)	H (no)
Self-perception	−0.12	0.20	−0.41*	−0.11	0.27	0.34	0.06	0.13
Affect regulation	−0.19	0.26	−0.32	−0.05	0.31	0.47*	0.05	0.19
Self-destruction	−0.11	0.28	−0.33	0.05	0.39*	0.42*	0.19	0.23
Dysphoria	0.08	0.31	−0.25	−0.02	0.17	0.25	0.09	0.17
Loneliness	−0.01	0.33	−0.31	0.02	0.45*	0.38*	0.13	0.04
Hostility	0.32	0.42*	0.05	0.18	0.42*	0.10	0.43*	0.13
Intrusions	−0.17	0.10	−0.37*	−0.12	0.31	0.29	0.09	0.06
BSL total score	−0.03	0.32	−0.34	0.00	0.41*	0.41*	0.19	0.18

*A* (*ne*) angry prime vs. neutral prime baseline, *A* (*no*) angry prime vs. no facial expression baseline, *H* (*ne*) happy prime vs. neutral prime baseline, *H* (*no*) happy prime vs. no facial expression baseline.

^a^Experimental condition.

* *p* < 0.05.

The subscales for self-destruction, loneliness, hostility, as well as the total score of the BSL were positively correlated with the latency difference score for the masked angry prime condition (neutral baseline condition). These correlations suggest that increased tendencies of self-aggression, hostility, and social isolation in BPD patients were related to response slowing due to masked angry faces. Similarly, the subscales for affect regulation, self-destruction, and loneliness, as well as the total score of the BSL, were positively correlated with the latency difference score based on masked angry primes (no facial expression baseline). Finally, there was a positive correlation between the subscale Hostility and the latency difference score based on masked happy primes (neutral face baseline).

## Discussion

In our study, automatic evaluative shifts and response delays owing to masked facial affects were investigated in patients with BPD and compared to healthy individuals. We examined also the relationship of affective priming effects with specific borderline symptoms and comorbidity. In our experiment, significant evaluative shifts due to masked angry facial expression were obtained for the total sample regardless of the baseline condition (neutral and no facial expression), which indicates that participants judged neutral mask faces more negatively when preceded by angry faces than when the neutral mask faces were preceded by neutral or no facial expression primes. Thus, our study provides evidence of valence-congruent evaluative shifts with respect to angry faces. Both masked angry and masked happy primes caused a significant delay in responses compared to neutral and no facial expression primes. It appears that a masked affective face compared to a neutral expression face slowed response time. This pattern of effects is similar to that found in emotional Stroop tasks [33]. Moreover, it has been repeatedly demonstrated that task-irrelevant affective information, regardless of valence, is automatically evaluated and attracts involuntary attention [34, 35]. Accordingly, it is assumed that automatic attention allocation facilitates further and more in-depth processing of relevant stimuli to guide adaptive behavior [33].

According to our results, BPD patients do not differ from healthy individuals in the automatic processing of angry and happy facial expressions. There is no evidence of a general automatic hypersensitivity (or hyposensitivity) for affective facial expressions in BPD patients with respect to influencing affective judgments and response speeds. Thus, our hypotheses that BPD patients’ evaluative judgments are more negatively affected by masked facial anger expressions and that their evaluative decisions are more disrupted or slowed by angry expressions than those of healthy persons were not confirmed. It appears that the automatic processing of negative facial affects is unimpaired in BPD patients. According to a recent meta-analysis [22], BPD patients are not characterized by global abnormalities or deficits in the recognition of negative facial affects at a controlled or conscious processing level. However, BPD patients might demonstrate enhanced learning of facial affect recognition after becoming familiar with people’s specific expressive characteristics compared to healthy individuals [36]. Against this background, it can be concluded that there are no indications of general abnormalities in the recognition of negative facial affects at a controlled and automatic processing stage in patients with BPD. In the whole sample, we further found no evidence for an enhanced initial allocation of attention to negative facial expressions as reported in a previous study [37]. Our findings are not consistent with those of Dyck et al. [38], which suggested a selective deficit in the rapid and direct discrimination of negative and neutral facial expressions in BPD patients, as the BPD patients in our study exhibited negative evaluative priming (due to angry facial expression). This means that they were apparently able to automatically read negative and neutral valences of masked prime faces and integrate this information into their judgments of subsequent expressions.

Previous research examining involuntary distraction or implicit processing in BPD patients showed a general attentional bias for negative disorder-related subliminal words [39] and a decreased capacity for automatic inhibition of irrelevant negative lexical stimuli in BPD patients [40]. Thus, it seems that individuals with BPD might manifest an enhanced involuntary allocation of attention to disorder-specific lexical information, but not to threatening (i.e., angry) facial expressions. To our knowledge, no work, to date, has been conducted on the automatic processing of facial affect in patients with BPD, which is surprising because, in general, affects are involuntarily elicited and emerge without conscious effort [41]. Using an emotional Stroop task, Arntz et al. [42] were the first to investigate vigilance to briefly presented (below the threshold of awareness) BPD-specific negative words, but found no indication of increased automatic sensitivity of BPD patients to disorder-specific lexical information compared to healthy individuals. These null findings could be due to the short presentation times and the small sample size.

Neither in our total sample nor in our patient sample were there correlations between evaluative priming scores and demographic variables, intelligence, depression, or trait anxiety. In the patient sample, intelligence was found to be inversely related to latency difference scores in the happy face condition, and depression was positively correlated with latency difference scores in the angry face condition. This suggest that attention allocation to masked happy faces could be more pronounced in patients with low intelligence and that allocation of attention to masked angry faces could be more pronounced in patients with high depression. More importantly, corroborating our assumption, the presence of comorbid anxiety disorders was found to be related to more evaluative shifting owing to masked happy primes regardless of baseline—neutral or no facial expression condition. Thus, consistent evidence was obtained showing that comorbid anxiety disorders are associated with stronger positive priming based on happy faces. Recently, Paul et al. [21] observed in a sample of healthy individuals that traits of social anxiety are positively related to priming effects due to masked happy faces. It has further been noted that happy or smiling faces can be interpreted as expressions of affiliation as well as expressions of threat by virtue of their association with dominance and devaluation [43]. Accordingly, the presence of comorbid anxiety in individuals with BPD might heighten sensitivity to subtle positive facial signals and increase its influence on subsequent evaluative processes.

Moreover, it was observed that BPD patients with obsessive–compulsive and paranoid personality disorders evaluated neutral masks preceded by angry primes as more positive than patients without these personality disorders. It appears that the presence of a comorbid obsessive–compulsive or paranoid personality disorder led to prime valence-incongruent shifts, thus suggesting that angry facial expressions might have been perceived as positive by BPD patients suffering from these personality disorders even though the expression denotes danger or threat. Correlation analyses based on latency difference scores indicated associations between response delay due to angry faces and the presence of comorbid affective disorder and paranoid personality disorder, such that both of these comorbid disorders might be related to an enhanced spontaneous allocation of attention to threatening facial expressions in BPD patients. It is not surprising that patients characterized by pervasive distrust and suspiciousness of others exhibited a heightened automatic vigilance for social threat. We found no correlations in the present study between affective priming or latency difference scores with the total number of comorbid Axis I or Axis II disorders.

Our correlation analyses further revealed only a few relations of borderline symptomatology with evaluative priming scores, but a larger number of associations with latency difference scores. In our study, the symptom hostility was associated with less negative priming due to masked angry faces. That is, at least in part, our data confirm the hypothesis that the hostility of BPD patients is related to less affective priming (i.e., evaluative shifts). In a previous study [20], interpersonal antagonism was found to be associated with impaired affect recognition in BPD patients. Furthermore, the symptoms of intrusions and difficulties in self-perception were accompanied by less positive priming owing to masked happy faces. Regardless of baseline—neutral and no facial expression condition—the symptoms of loneliness and self-destruction, as well as the total score of the BSL, were associated with response slowing due to masked angry faces. It appears that BPD patients suffering from social isolation and self-aggression are characterized by an enhanced allocation of attentional resources toward threatening facial expressions. High sensitivity to subtle social threat signals might be a contributing factor to social withdrawal and may reinforce beliefs that others are malevolent and hostile.

In sum, the present correlation analyses have revealed several associations of response slowing to masked angry faces with the presence of comorbid disorders and specific borderline symptoms. BPD patients with comorbid depressive disorder, paranoid personality disorder, and those suffering from social isolation and self-aggression seem to involuntarily allocate more attention resources to angry facial expressions than other patients. Patients suffering from these comorbid disorders or symptoms might be especially sensitive to interpersonal threat signals of minimal intensity. This finding should be kept in mind when interacting with depressed, paranoid, socially withdrawn, or auto-aggressive patients with BPD.

Furthermore, neuroimaging research has shown that BPD patients manifest an exaggerated amygdala response to affective facial expressions [44, 45]. The amygdala is critically involved in the detection of biologically relevant, threatening, and ambiguous stimuli [46, 47] and in the modulation of vigilance to augment subsequent information processing throughout the brain [48]. Ripoll et al. [49] formulated a neurobiological model of empathic dysfunction in BPD patients to determine whether heightened amygdala response in individuals with BPD favored the detection of affective salience, and especially facial affects. It could also contribute to automatic attunement to other persons’ feeling states and lead to emotional contagion, especially with respect to negative affects. There is consistent evidence that depression increases the responsivity of the amygdala to masked negative faces [50–52]. In an fMRI study using a priming paradigm, a positive correlation was observed between amygdala activation and evaluative latency in response to masked sad facial expression [53]. Thus, it appears plausible to link delays in the processing of masked negative facial affects to amygdala reactivity. An enhanced allocation of attention to angry expressions could contribute to the generation of negative affective reactions and, thus, to affective instability in BPD patients.

Interestingly, in our study, after the priming task BPD patients reported having seen more negative affect expressions (i.e., faces expressing sadness and fear) that had not been presented during the experiment compared to healthy individuals. This response behavior could indicate a bias toward the perception of threat-related or negative facial expression in BPD patients and is consistent with previous research that suggests a misattribution of negative affects to faces depicting neutral or ambiguous expressions [10, 22]. According to Daros et al. [54], especially subtle expressions of negative affect might be subjectively magnified by individuals with BPD.

We do not claim to have assessed subliminal affect processing in our study. However, insofar as the duration of face presentation was extremely brief, our task should have measured automatic responses to facial affects. It must be acknowledged that automaticity with regard to information processing is not a unitary construct, but rather diagnosed by considering the presence of different features such as unintentional, uncontrollable, unconscious, efficient, and fast [55]. However, not all features must be present to assume automaticity [56]. In our study, the processing of facial affect expression should have been automatic in the sense of being fast and unintentional. Study participants had no conscious intent to process the affective faces, and the perception of affective facial expression occurred under conditions of distraction or inattention. Moreover, the processes elicited by the affective faces should have been fast, as affective primes were displayed for only a thirtieth of a second and were immediately followed by a mask.

The fact that BPD comorbidity rates for Axis I and Axis II disorders were high in our sample is a point of controversy. Therefore, it is necessary to conduct further research with other patient samples and examine men as well before stronger conclusions about automatic perception of facial affect in BPD patients can be drawn. However, BPD samples without Axis I co-occurring disorders are atypical and “pure” BPD clinical samples are rare.

Inconsistencies in findings of previous studies on facial affect processing in BPD patients and, in part, weak effects in the present study may stem from extreme changeability of affect perception in BPD. The processing of affective information could vary between BPD patients and over time, for example, as a function of the present social situation, current mood, actual comorbid disorders, or attachment style. Thus, a variety of situational and dispositional factors must be considered when examining both automatic and controlled facial affect processing in individuals with BPD.

## Conclusions

In the present affective priming experiment, no evidence was found of a general automatic hypersensitivity for affective facial expression in individuals with BPD with respect to influencing affective judgments and response speed. The patient group did not differ from the healthy control group in evaluative shifts and response delays caused by masked facial affects. It can be concluded that there are no indications of abnormalities in the automatic recognition and processing of negative or positive facial affects in BPD patients. Comorbid disorders and specific borderline symptoms, however, were found to be associated with evaluative shifts and response delays. According to our data, patients with comorbid anxiety disorders were more susceptible to the influence of happy expressions on judgment processes at an automatic processing level. Moreover, comorbid depressive disorder, paranoid personality disorder, and symptoms of social isolation and self-aggression may enhance automatic attention allocation to threatening facial expressions in individuals with BPD. Increased automatic vigilance for social threat stimuli might contribute to affective instability and interpersonal problems in specific patients with BPD.
